# Using Live Imaging and FUCCI Embryonic Stem Cells to Rank DevTox Risks: Adverse Growth Effects of PFOA Compared With DEP Are 26 Times Faster, 1,000 Times More Sensitive, and 13 Times Greater in Magnitude

**DOI:** 10.3389/ftox.2021.709747

**Published:** 2021-11-30

**Authors:** Mohammed Abdulhasan, Ximena Ruden, Yuan You, Sean M. Harris, Douglas M. Ruden, Awoniyi O. Awonuga, Ayesha Alvero, Elizabeth E. Puscheck, Daniel A. Rappolee

**Affiliations:** ^1^ Department of Ob/Gyn, CS Mott Center for Human Growth and Development, Reproductive Endocrinology and Infertility, Wayne State University School of Medicine, Detroit, MI, United States; ^2^ Reproductive Stress 3M Inc, Grosse Pointe Farms, Detroit, MI, United States; ^3^ Program for Reproductive Sciences and Department of Physiology, Wayne State University School of Medicine, Detroit, MI, United States; ^4^ Department of Environmental Health Sciences, School of Public Health, University of Michigan, Ann Arbor, MI, United States; ^5^ Invia Fertility Clinics, IL, Chicago, United States; ^6^ Institutes for Environmental Health Science, Wayne State University School of Medicine, Detroit, MI, United States; ^7^ Department of Biology, University of Windsor, Windsor, ON, Canada

**Keywords:** HTS, high through put screening, perfluorinated alkylated substances, phthalates, developmental toxicity, embryonic stem cell, stress, viable cell count studies

## Abstract

Fluorescent ubiquitination-based cell cycle indicator (FUCCI) embryonic stem cells (ESCs), which fluoresce green during the S-G2-M phases, generate an S-shaped curve for the accumulation of cells during normal stemness (NS) culture with leukemia-inhibitory factor (LIF). Since it was hypothesized that a culture of ESCs was heterogeneous in the cell cycle, it was expected that increased S-G2-M-phases of the cell cycle would make an S-shaped curve parallel to the accumulation curve. Unexpectedly, it was observed that the fraction of FUCCI ESCs in green decreases over time to a nadir at ∼24 h after previous feeding and then rapidly enters S-G2-M-phases after medium change. G1 delay by infrequent medium change is a mild stress, as it does not affect growth significantly when frequency is increased to 12 h. Perfluoro-octanoic acid (PFOA) and diethyl phthalate (DEP) were used as examples of members of the per- and polyfluoroalkyl substances (PFAS) and phthalate families of chemicals, respectively. Two adverse outcomes were used to compare dose- and time-dependent effects of PFOA and DEP. The first was cell accumulation assay by time-lapse confluence measurements, largely at Tfinal/T74 h. The second was by quantifying dominant toxicant stress shown by the suppression of mild stress that creates a green fed/unfed peak. In terms of speed, PFOA is 26 times faster than DEP for producing a time-dependent LOAEL dose at 100 uM (that is, 2 h for PFOA and 52 h for DEP). PFOA has 1000-fold more sensitive LOAEL doses than DEP for suppressing ESC accumulation (confluence) at day 3 and day 2. There were two means to compare the magnitude of the growth suppression of PFOA and DEP. For the suppression of the accumulation of cells measured by confluence at Tfinal/T74h, there was a 13-fold suppression at the highest dose of PFOA > the highest dose of DEP. For the suppression of entry into the cell cycle after the G1 phase by stress on day 1 and 2, there is 10-fold more suppression by PFOA than DEP. The data presented here suggest that FUCCI ESCs can assay the suppression of accumulated growth or predict the suppression of future growth by the suppression of fed/unfed green fluorescence peaks and that PFOA’s adverse effects are faster and larger and can occur at more sensitive lower doses than DEP.

## Introduction

Comprehensive *in vitro* developmental toxicity (DevTox) assays are controversial, and the current standard is *in vivo* rodent gestational testing for the Extended One-Generation Reproductive Toxicity Study (Beekhuijzen et al., 2016). With the proliferation of new drugs and commercial compounds in development and the existing drugs and compounds with insufficient DevTox data for regulatory risk assessment, NIEHS, EPA, and the National Academy of Sciences (NAS) have formulated strategies for high-throughput screens (HTS) to mediate massive *in vitro* testing (Krewski et al., 2010; Parish et al., 2020) to inform or refine *in vivo* DevTox testing. Previous ESC tests for cytotoxicity, cardiotoxicity (Genschow et al., 2000; Genschow et al., 2002), and metabolomic outcomes (Zurlinden et al., 2020) have been validated. Many tests of pluripotent stem cells pre-differentiate these cells to other cell types, but the studies mentioned above immediately assess toxicant dose-dependent effects on stem cells cultured as stem cells.

Cell-based HTS are an important part of drug discovery and helps in assessing the risk of drug and environmental compound exposure (Macarron et al., 2011). An important concept for DevTox *in vitro* HTS is that the cells used retain the developmental, molecular, and cellular strategies to respond to toxicant stress in the same way that they used to in the milieu of the early *in vivo* post-fertilization embryo they were isolated from (Rappolee et al., 2012; Puscheck et al., 2015a; Yang et al., 2017a). Embryonic and placental trophoblast stem cells (ESCs and TSCs, respectively) are isolated from the early embryo just prior to implantation into the uterus. *In vitro,* these stem cell lineages do seem to emulate the strategies needed for the survival of the stressed embryo, *in vivo*.

The early embryo at implantation has ESCs and TSCs, and both lineages enter the unique period of exponential growth as the two lineages arise, and this period lasts for about 10 days during mouse gestation and for 2–3 weeks in human gestation. Exponential growth supports rapid embryonic expansion and sequential differentiation of the first few differentiated lineages from either ESCs or TSCs. The first differentiated lineages to arise are essential for the survival of the implanted embryo. *In vivo*, if sufficient parenchymal function is produced, the embryo survives. Stress from medical (IVF) or toxicological sources should decrease stem cell growth and force first lineage differentiation to compensate for insufficient cell number and parenchymal function.

Monitoring cell growth in every well of a high-throughput screen (HTS) is not possible by the naked eye, and it is necessary to use an automated live imager or microplate reader to assay the cell number, nuclear number, or confluence in every well of the HTS plate (Boutros et al., 2015). Growth reduction by dose-dependent stress is an important basis to integrate other downstream outcomes such as lineage imbalance or epigenetic deviation which are proportional to growth reduction (Puscheck et al., 2015b). However, the growth effects of stress are complicated as rapidly growing cells with zero stress dose may become contact-inhibited and confluent at Tfinal which is needed to optimize the detection of other developmental outcomes. Since Tfinal cell accumulation assays require the completion of cell division, they are “lagging indicators” of previous stress effects. The reliability of growth reduction assays is marred by the confounding decrease in cell growth rates in lower stress-dosed wells that reach confluence earlier.

One “leading indicator” would be the use of a fluorescent ubiquitination-based cell cycle indicator (FUCCI) to test for cells leaving the G1 phase and entering the S-G2-M-phase, reported by green fluorescence of the Geminin–Azami Green fusion protein, to eventually complete the cell cycle (Zielke and Edgar, 2015). Hypothetically, FUCCI green cell cycle progression would be diminished by stress on the first day of exposure, predicting a decrease in confluence and cell number or nuclear number by the same stress and dose at Tfinal.

PFOA and DEP represent large families of environmental toxicants, that is, PFAS and phthalates, respectively. Phthalates are esters of phthalic acid which are used in cosmetics, personal care products, and polyvinyl chloride (PVC) plastics (Dutta et al., 2020). Since phthalates are not covalently bonded into plastics, they enter the environment and then the body by absorption, ingestion, or inhalation. Specifically, diethyl phthalate (DEP) is a low molecular weight (LMW) phthalate often used as a solvent in the manufacture of personal care products. DEP has previously been shown to increase neural markers at non-cytotoxic doses but slows mouse embryonic stem cell growth (Yin et al., 2018). PFOA is a surfactant used in many industries, such as textiles and floor wax synthesis and in fire-fighting foam and sealants (Lindstrom et al., 2011). PFOA is a weak embryotoxic chemical often acting as an endocrine disruptor as defined by embryonic stem cells (ESCs) but can have additive but not synergistic effects with BPA (Zhou et al., 2017). Although structurally similar, PFOS is synergistic with BPA. This is relevant because real-world stressors are episodic, repeated simultaneously and synergistically, intrinsic, and environmental (Awonuga et al., 2013; Puscheck et al., 2015a). During gestational exposure, PFOA increased maternal weight but decreased fetal weight (Blake et al., 2020) PFOA is unique amongst the PFAS family in causing early first trimester miscarriage (Wikström et al., 2021), which can be modeled using embryonic and placental stem cells (Puscheck et al., 2015a; Yang et al., 2017a). These families of toxicants incur large health costs to Americans in general and to pregnant women and their embryos/fetuses/placentas specifically. Since they represent thousands of individual toxicants, HTS are needed to estimate DevTox exposures.

Traditionally each toxicant is assigned no and lowest adverse effect levels (NOAEL and LOAEL, respectively) and half maximal toxic dose for each effect measured (IC_50_), but other points of departure have been used more recently. One such point of departure that we have also used is the benchmark dose (lower confidence limit), known as the BMDL. In addition, we have developed the concept of a “direness index” to match the “stimulation index” (Yang et al., 2017b; Puscheck et al., 2018). We used the direness concept and dose-dependent kinetics of stressed stem cell responses reported by the live imager, to develop a separate predictive outcome for growth suppression.

Here, we tested FUCCI ESCs for the effects of leading and lagging indicators of growth in a live imager and reported dose-dependent suppression of cell cycle commitment by PFOA and DEP as examples of the PFAS and phthalate families, respectively. We also reported an unanticipated FUCCI S-G2-M-phase report of a time delay in the G1phase before the S phase resulting in a green nadir. The nadir is rapidly reversed into a green fluorescence peak by medium change. Here, we use toxicant dose-dependent suppression of this fed green peak as a sensitive indicator of future growth suppression, free of the confounding variable of contact inhibition of growth in low stress-dose responders that reach confluence at the earliest.

## Materials and Methods

### Materials

FUCCI mESCs were a kind gift from Dr Pierre Savatier (Coronado et al., 2013). Pdgfra-GFP mESCs were validated in a previous report (Li et al., 2019) (Mouse 129S4-derived AK7 ESCs that express the transgenic fluorescent protein (GFP) that report Pdgfra and extends expression) and was a kind gift from Dr. Anna-Katerina Hadjantonakis (Sloan Kettering Institute, New York, NY). The Pdgfra-GFP cell line is heterozygous for a knock-in H2B-GFP fusion gene replacing the first two immunoglobulin domains of the Pdgfra ligand-binding domain, enabling GFP expression in nuclear histones (Kanda et al., 1998) that enables cell counts and the analysis of ESC differentiation to ExEndo (Hamilton et al., 2003; Artus et al., 2010). And Oct4-GFP mESCs were a kind gift from Promega (Boiani et al., 2002). DMEM was obtained from Sigma (Sigma, St. Louis, MO Cat# D6546, Gibco). Glutamax and sodium pyruvate supplement solutions were obtained from Life Technologies (Grand Island, NY). ESC-qualified EmbryoMax fetal bovine serum, 0.1% gelatin solution, and ESGRO™ Mouse LIF medium supplement were obtained from EMD Millipore (Billerica, MA). MEM non-essential amino acid solution, sorbitol, 2-mercaptoethanlol, and other chemicals were obtained from Sigma (St. Louis, MO). Perfluoro-octanoic acid (PFOA) and diethyl phthalate (DEP) were purchased from Sigma-Aldrich (Cat# 171,468 and 524,972, respectively). The parental 129S4/SvJae ESC (D3 mouse strain) was purchased from ATCC (Manassas, VA) (Doetschman et al., 1985a).

## Methods

### Embryonic Stem Cell Culture

FUCCI, Pdgfra-GFP, and Oct4-GFP mouse embryonic stem cells were cultured as described previously (Li et al., 2016a; Li et al., 2016b; Li et al., 2019). All ESCs were optimized at the passage for exponential growth during the stimulus period, which began 18 h after passage at 24% confluence. Germline-competent mESC-D3 cells (ATCC, Manassas, VA) from Doetschman et al. (1985b) were cultured in the absence of feeder cells in DMEM supplemented with 15% mESC-screened FBS, 2 mM L-glutamine, 1 mM sodium pyruvate, 1 mM nonessential amino acids, 0.1 mM 2-mercaptoethanol, and 1000 U/mL murine LIF on 0.1% gelatin-coated dishes at 37⁰C in humidified air with 5% CO_2_ (Masui et al., 2008). mESCs were cultured overnight after passaging before stimulation with sorbitol-positive control stress or experimental stressors PFOA and DEP. The osmolality of ESC media with and without addition of 200–300 mM sorbitol was determined previously (Slater et al., 2014).

### Fluorescent 96-Well Reading as a High-Throughput Screen for Fluorescent Ubiquitination-Based Cell Cycle Indicator mESCs

FUCCI mESCs were cultured on black and clear-bottom 96-well plates overnight to reach ∼ 25% confluence in a Fisher dual CO_2_ incubator with ambient oxygen and 5% CO_2_ at 37°C (Thermofisher Scientific, HeraCell Vios 160i dual CO_2_ incubator, Waltham, MA). Then, the toxicants were added at Tzero (∼18 h after passage) and the FUCCI mESCs were cultured in a Sartorius IncuCyte Zoom system (Essen Biosciences Inc., Ann Arbor, MI) for 72 h. The images were acquired by 4x objective (Nikon APO λ, OFN25) in the IncuCyte Zoom microscope with a dual-color model which can detect the green signal (excitation: 440–480 nm, emission: 504–544 nm) every 2 h for every well in the 96-well plates. The IncuCyte Zoom incubator was set up with ambient oxygen and 5% CO_2_. The acquisition software was provided by IncuCyte Zoom (Sartorius, version 2016 B). The whole-well image was taken at both phase (auto exposure) and GFP channel (800 ms). The medium was changed every 24 h except for the experiment where the medium was changed every 12 h, but there was some variability in this schedule. The cells were treated with seven concentrations of toxicants from 0 to 1, 10, and 100 nM and 1, 10, and 100 uM in LIF + media for three days.

### Statistical and Graphical Analyses

Initial data analysis was conducted using acquisition software and was provided by IncuCyte Zoom (Sartorius, version 2016 B) This software analyzed the pictures to calculate the confluence and total green object integrated intensity of each well at each timepoint. Total green object integrated intensity is the sum of the intensities of the green objects. We calculated this stimulation index by, for each replicate, selecting the lowest nadir and single highest peak before and after medium change, respectively. The peak was chosen to be the highest value between T26–T36 or T50–T60, respectively (the highest value within 10 h after taking the reading immediately after the average feeding time). The trough was chosen to be the lowest point within 10 h preceding the chosen peak if the start of the peak was not easily seen. If the start of the peak was a sharp departure (the green vs time graph had an angle <120°), then the vertex of the sharp angle was chosen as the trough. Graphs were initially composed and formatted using MS Excel (MS Office 365) and final formatting was performed using Photoshop Elements Photoshop 13 Editor (Adobe systems incorporated, San Jose, Ca). Some initial statistics were conducted to estimate the LOAEL and IC_50_ value using GraphPad Prism 9.0 (GraphPad software, San Diego, Ca), and point or departure analysis was performed using benchmark dose software (BMDS) 3.2 (EPA, Research triangle park, NC). The analysis of time-dependent points of departure in this article is a simply the first significant adverse effect. But, we acknowledge that other people have used other time-dependent points of departure called “tipping points” of departure for time and magnitude that are more complex and report irreversible adverse effects (Shah et al., 2016; Saili et al., 2020).

***Benchmark dose modeling.*** The BMD is a point of departure defined as a dose that elicits a specified response and the BMDL is the lower limit at 95% confluence of the estimated BMD. The BMD and BMDLs for PFOA and DEP impacts on cell confluence were estimated using benchmark dose modeling software (BMDS) version 3.2 (USEPA). We selected a benchmark response of one standard deviation of the control samples, as recommended by the USEPA (Crump, 1995). We tested all models available in the default settings of BMDS, including linear, polynomial, exponential, and Hill models. The optimal model was selected based on the visual assessment of the dose–response curve( so that the experimentally derived curve and curve fit were most similar), lowest Akaike’s information criterion (AIC) (Akaike, 1974), and largest goodness-of-fit *p*-value (>0.1) (Dodge, 2010). For PFOA, while none of the models tested had a goodness-of-fit *p*-value of >0.1, the Hill model had the lowest AIC of the models tested and was used to estimate the BMD (484 nM) and BMDL (278 nM). For DEP, we chose the frequentist Exponential degree 4 v1.1 model because it has the lowest AIC and the highest *p*-value (though still <0.1).

## Results

### ESCs Do Not Grow Heterogeneously in the Cell Cycle but Show Feeding-Dependent Delay in the G1 Phase, Followed by Rapid Re-entry Into the S-G2-M-phase After Medium Change

**FIGURE 1:**
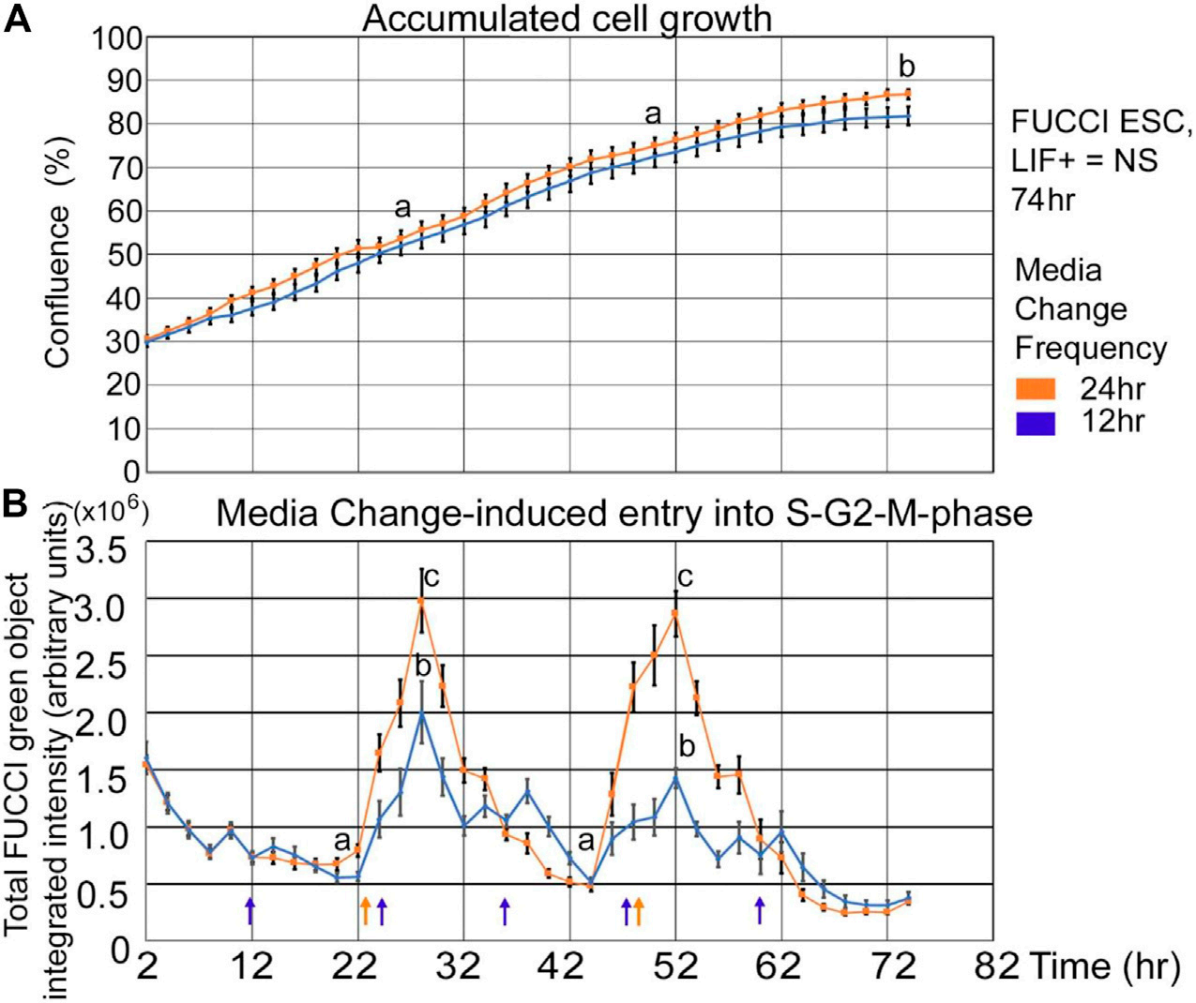
FUCCI ESCs accumulate in an S-shaped curve, but medium change reveals that cells accumulate in the green-negative state (G1) approaching a 24-h interval feeding and then rapidly re-entering the green S-G2-M-phase after medium change. FUCCI ESCs were passed into 96-well plates and 18 h later had the first medium change of NS/LIF + media at Tzero, placed in a live imager, were fed at 24 and 48 h, or 12, 24, 36, 48, and 60 h (shown by arrows in Panel 1B), and then assayed every 2 hours for confluence **(A)** or FUCCI total object green intensity **(B)** in an IncuCyte Zoom live imager. The times for medium change may vary by 2 hours. Error bars are s.e.m. from five independent biological experiments for 12 h and six independent experiments for 24h feeding frequency, respectively. In Panel 1A, (a) shows no significant difference between 12- and 24-h feeding at 24 and 48 h, but (b) shows significant difference at 74 h (*p* < 0.05). In Panel 1B, the nadirs (a) are significantly different than the peaks (b, c) for both feeding schedules (*p* < 0.0001). *N* = 26; 12-h feed: five biological experiments, *N* = 15.

Cultured pluripotent stem cells should grow exponentially after passage and be heterogeneous in the cell cycle. Stem cells in the early mammalian embryo are not synchronized in the cell cycle, in contrast to the high degree of synchronization of pre-gastrulation stem cells in frogs and fruit flies (Gilbert, 2010). People may use serum starvation and abnormal cell cycle synchronization to aid in preprogramming (Chen et al., 2012). Growth measured by confluence shows an S-shaped growth curve: In Figure 1A a live imager records confluence every 2 hours in five or six independent biological experiments for 12- and 24-h feeding frequency, respectively. And, as expected of normal embryonic stem cell culture cells under naïve pluripotency conditions with LIF, there is exponential growth. Standard ESC culture conditions require medium change every day to remove lactate from aerobic glycolytic. Warburg metabolism was used by stem cells to maintain good growth conditions and to feed the cells. ESCs found heterogeneously in the cell cycle seem like they would have an exponential increase in green cells with a line similar in shape and lower than the confluence line as shown in Figure 1A . But in Figure 1B, the number of cells in the S-G2-M phase reaches a nadir in the last few hours prior to the 24-h medium change and then increases in the hours following medium change. It should be noted that increase in green fluorescence in a single experiment varies from two–12 h after the nadir, although it is usually 6–8 h after the nadir. Also, the nadir is reached ∼two h before feeding, perhaps due to some daily cycle that ESCs go through.

It was hypothesized that insufficient medium change effects would be ameliorated by more frequent medium changes. Two effects were expected; that the peak amplitude would decrease and the growth rate would increase with more frequent feedings. In Figure 1B, it is shown that 12-h feeding does decrease the amplitude of the peak green number of cells entering the S-G2-M phase after medium change, but in Figure 1A there was no significant increase in cell growth mediated by more frequent medium changes.

Fold change in fed/unfed green peaks is greater for ESCs responding to 24-h medium change frequency than for 12 h.

**FIGURE 2:**
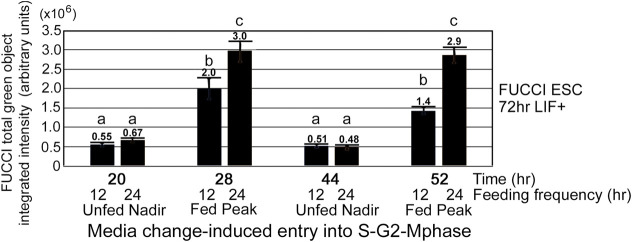
The stimulation index of 24-h feeding frequency peak is greater than the 12 h-feeding frequency peak. **(A)** FUCCI ESCs enter the S-G2-M phase after medium change. 24-h feeding frequency has more cells entering the S-G2-M phase after medium change than does 12-h feeding frequency. FUCCI ESCs were passed into 96-well plates and had first medium change of NS/LIF + media at Tzero, were fed at 24 and 48 h or 12, 24, 36, 48, and 60 h, and then assayed for FUCCI total object green intensity in an IncuCyte Zoom live imager at the times indicated. Statistical comparisons from five or six independent biological experiments for 12- and 24-h feeding frequency, respectively: a, b), a is not significantly different than b, but b at 44-h timepoint is a significantly lower nadir than b at 20-h timepoint (*p* < 0.01), c.) 12-h peak is significantly higher than 12-h nadir (*p* < 0.0001), and d.) 24-h feeding frequency peaks are significantly higher than 12-h frequency peaks (*p* < 0.05). Same N as shown in Figure 1.

As shown in Figure 1, cells are not heterogeneous in the cell cycle, and we analyzed the nadirs in total green object integrated intensity before and after medium change by calculating the fold change between the peak divided by the nadir. N = 26 for the 24-h feeding schedule, and N = 15 for the 12-h feeding schedule. Picking the nadir and peak for each replicate was performed to get the most accurate ratio as the timing of feeding and acquisition of green fluorescence may vary between experiments. More detail of picking peaks is shown in the Statistics subsection of the Materials and Methods section. To better understand the contributions of changes in nadirs and/or peaks to the changes in fold-change reported for 12- and 24-h medium change, we analyzed these component changes in Figure 2A . The nadirs from 12- to 24-h feeding frequencies were not significant, but the peaks for both frequencies were higher than the nadirs, and the 24-h peak was significantly higher than the 12-h peak.

**FIGURE 3:**
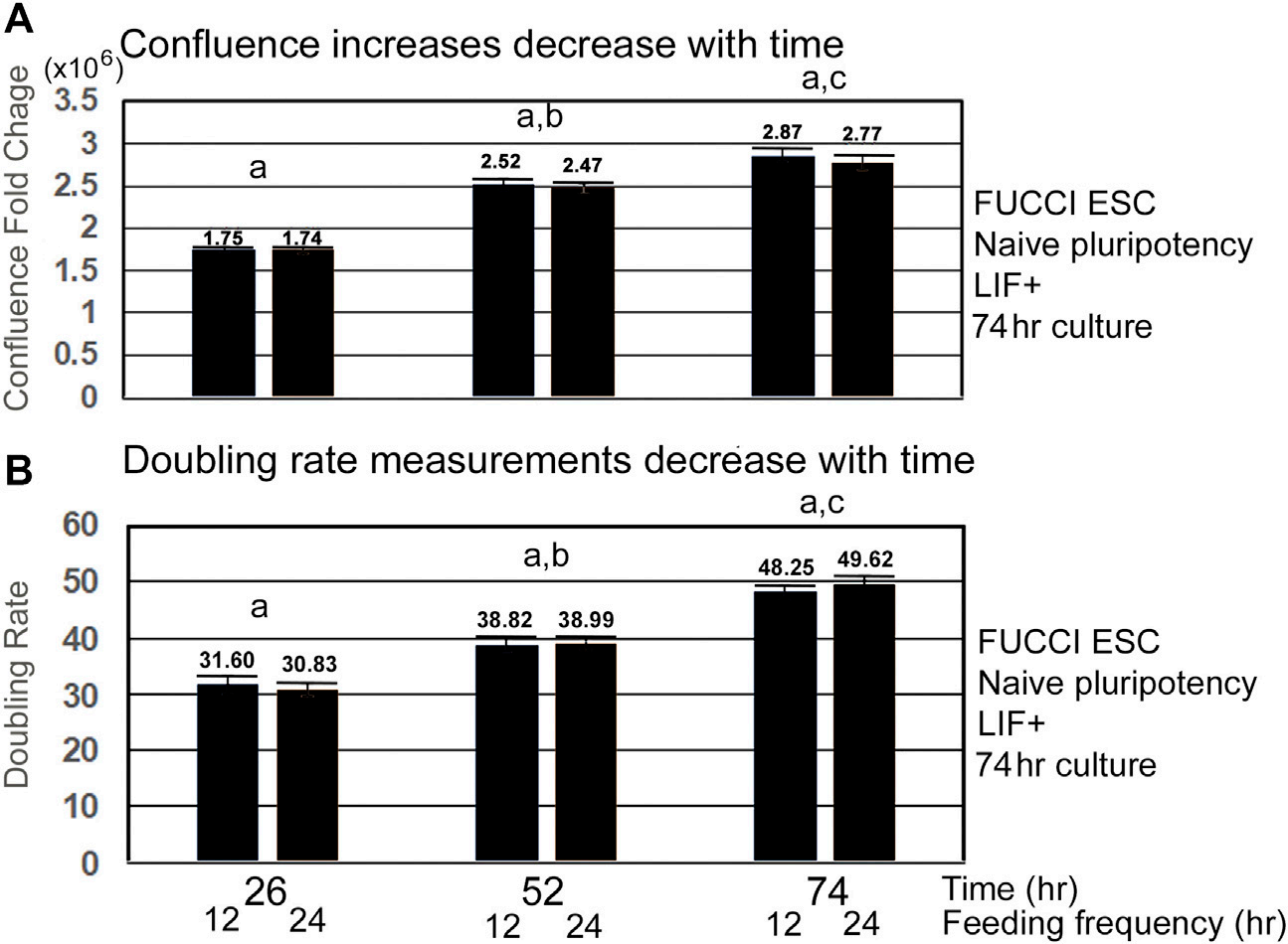
Velocity of increase in confluence decreases with time, and doubling rates get slower with time as confluence and contact inhibition increases. FUCCI ESCs enter the S-G2-M phase after medium change during NS culture with LIF. FUCCI ESCs were passed into 96-well plates and had first medium change of NS/LLIF + media at Tzero, were fed at 24 and 48h or 12, 24, 36, 48, and 60 h, and then assayed for confluence in an IncuCyte Zoom live imager. **(A)** shows the fold change whereas **(B)** shows the doubling rate of confluence from the stated time to 2 h. (a) There is no significant difference in fold change or doubling rate for the different feeding schedules. (b) At 52 h, FC and doubling rates are significantly slower than those for 26 h (*p* < 0.005). (c) Time 74 FC and doubling rates are significantly different than those for Time 52 (*p* < 0.05 for FC, *p* < 0.0001 for doubling rates). Five independent biological experiments from 12-h feeding frequency and six independent biological experiments for 24-h feeding frequency with paired replicate wells in each experiment for each dose and stimulus were used for statistical analysis. Same N as shown in Figure 1. Confluence at T68 was used instead of T74 for datasets without T74.

As confluence is reached and contact inhibition increases, the velocity of the lagging indicator of growth measurements decreases. Lagging indicators for growth, such as confluence and doubling rate decrease to a plateau value or to zero, respectively, as cells approach 100% confluence and become contact-inhibited (Figures 3A,B). Interestingly, the doubling rate is shortest, indicating fastest growth rate only on the first day, suggesting this would be a good period to assay for instantaneous changes in the growth rate caused by increase in toxicant stress doses.

**FIGURE 4:**
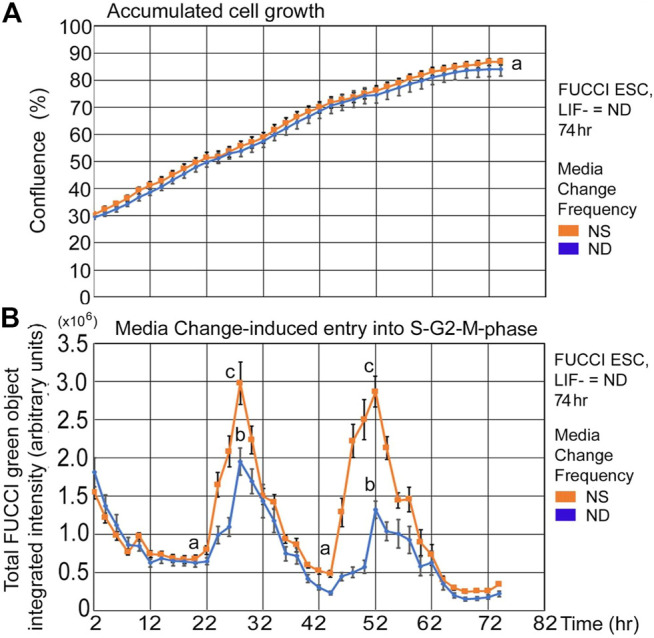
FUCCI ESCs have similar rates of confluence increase and fed/unfed induced entry into the S-G2-M-phase during normal differentiation as in normal stemness culture. FUCCI ESCs were passed into 96-well plates and had medium change of either NS/LIF + or ND/LIF- media at Tzero, 24, and 48 h and then assayed for confluence and FUCCI total object green intensity in a IncuCyte Zoom live imager. **(A)** Cells were measured in time-lapse every 2 hours for confluence in the live imager. **(B)** FUCCI ESCs were fed at 24-h frequency and assayed for green fluorescence in time-lapse every 2 h from 2–74 h. Statistical comparisons from six independent biological experiments: Panel 4A **:** no significant difference between confluence at any timepoint and Panel 4B: Nadirs (a) are significantly different than peaks (*p* < 0.0001). Peaks for NS (c) are significantly larger than for ND (b) (*p* < 0.0001). NS: N is described in Figure 1; ND: six biological experiments, N = 17.

FUCCI ESCs undergoing pluripotency restriction (differentiation) due to LIF removal, have a similar rate of confluence increase and similar fed/unfed S-G2-M-phase increases compared with cells in stemness culture. After LIF removal, ESCs slowly lose stemness and pluripotency-maintaining transcription factors, but the culture conditions here support similar time-dependent increases in confluence (Figure 4A). Similarly, instantaneous entry into the S-G2-M-phase showed a sharp increase from the unfed to fed states, though ND culture has a shallower increase than NS (Figure 4B).

**FIGURE 5:**
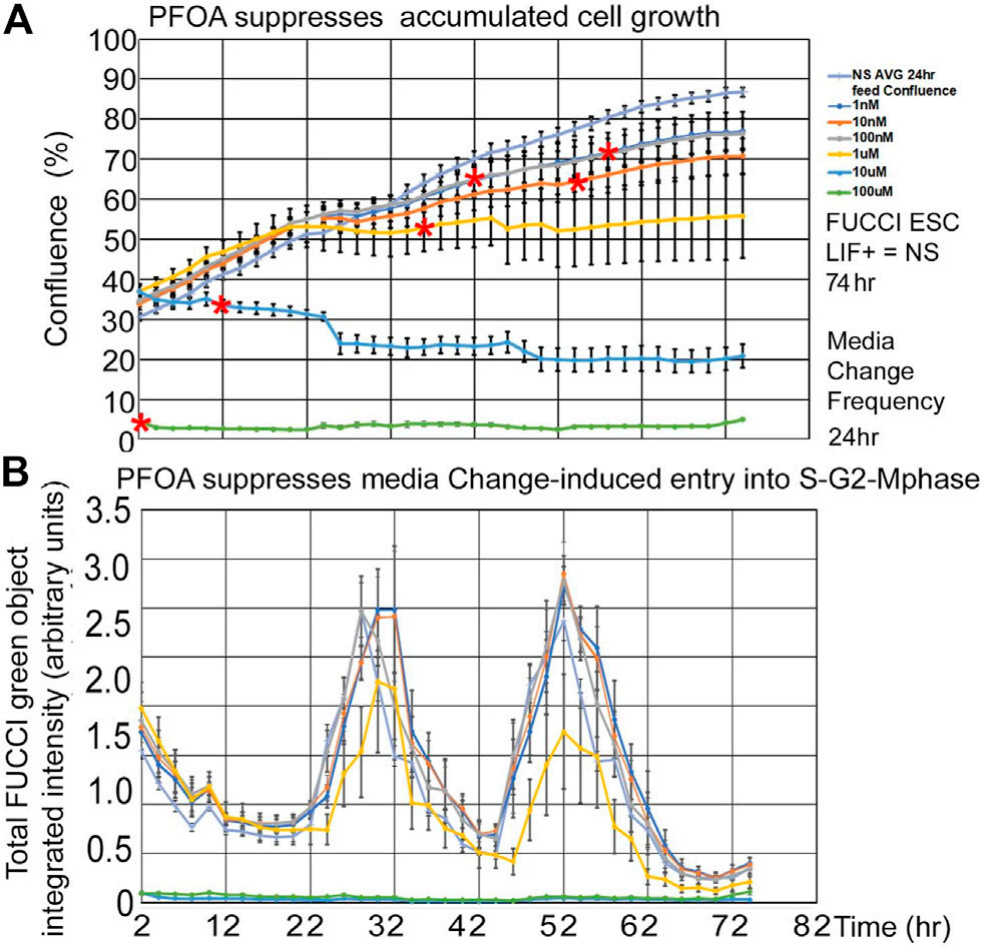
Three highest PFOA doses suppress entry into the cell cycle, but only the two highest doses suppress the lagging phase accumulated growth measured by confluence. FUCCI ESCs were cultured in a live imager with 2-h time-lapse for confluence and green fluorescence for six doses of PFOA from 1–10–100nM and 1–10–100uM added to NS media. **(A)** Cells were measured in time-lapse every two hours for confluence in the live imager. As the cells grow for a longer period, the effect of the doses increases, causing the LOEL to be a lower dose. Red asterisks show the timepoint where confluence becomes significantly different than 0 nM PFOA/NS (the direness points of departure). **(B)** Green cell cycle entry into the S-G2-M-phase was measured simultaneously with confluence measurements in A and after feeding at day 1 and 2. In general, the green peak decreases as the dose of PFOA increases. NS (0 dose): N is described in Figure 1. PFOA: four biological experiments. N = 8 for each dose except 100 uM where N = six or eight depending on the timepoint.

PFOA suppresses fed/Unfed peak entry into the S-G2-M-phase of the cell cycle. Line graphs of time lapse data show that PFOA suppresses accumulated growth as a lagging indicator measured by confluence (Figure 5A) and suppresses entry into the S-G2-M phase after medium change as a leading indicator (Figure 5B ) predicted during ESC culture with LIF.

**FIGURE 6:**
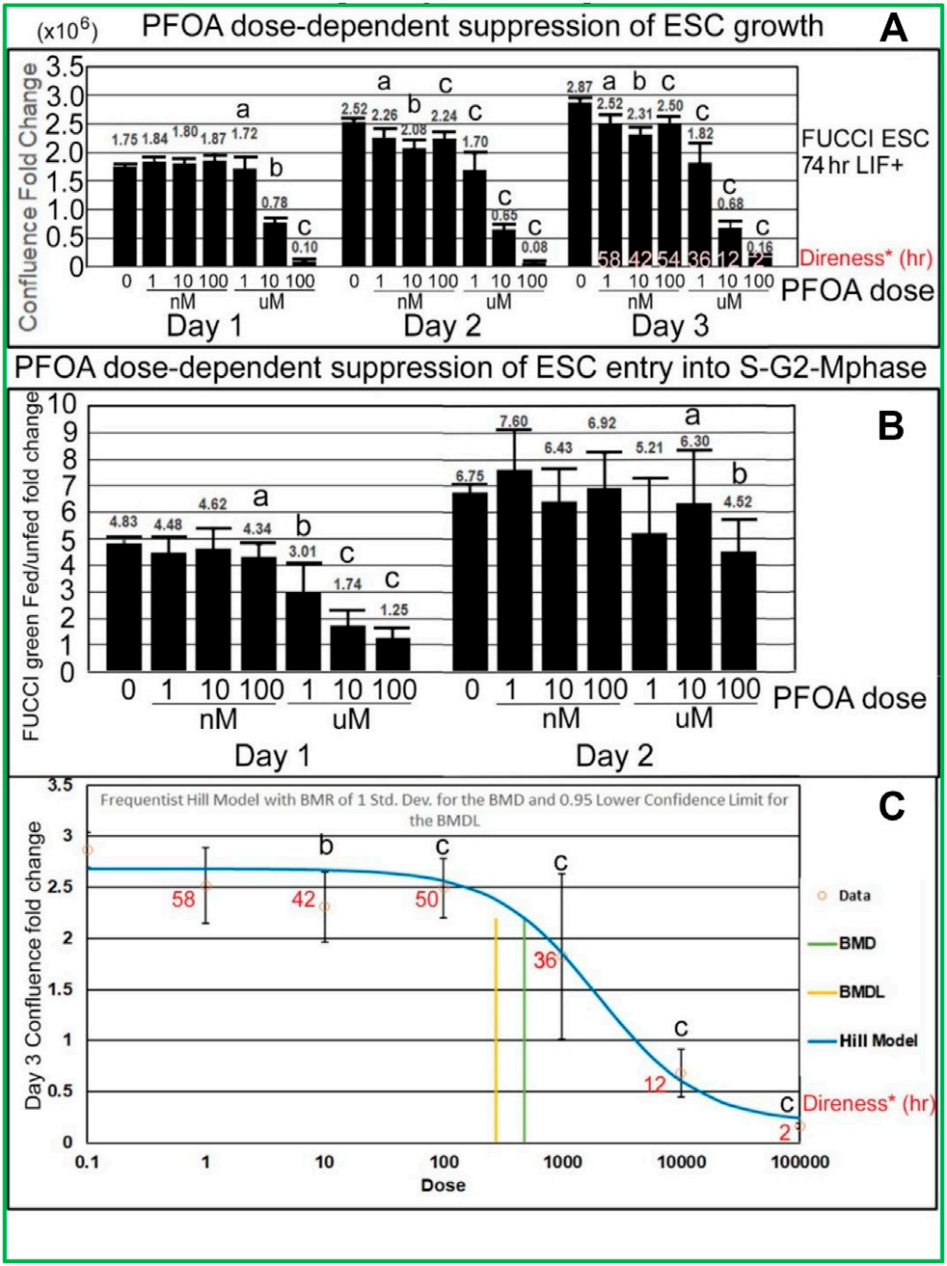
PFOA decreases accumulated cell confluence for all three culture days, but instantaneous increase in fed/unfed entry into the S-G2-M-phase shown by green peak fluorescence was more sensitive on day 1 than day 2. **(A)** Confluence fold change from the indicated time to time zero for different doses of PFOA are shown (T26/T0, T52/T0, and T74/T0). As data of the confluences of the wells before the first feeding of the toxicant are not known, T0 was used (2 h after the first feeding) for NS. As the cells grow for a longer period, the effect of the doses increases, causing the LOEL to be a lower dose. The direness points of departure from Figure 5A are shown in the day 3 bars. **(B)** The amount of green fluorescence at the peak after feeding and the nadir before feeding is rationated. In general, the green fold change decreases as the dose of PFOA increases. **(C)** Day 3 confluence FC from A was analyzed, using the Benchmark dose as point of departure. Direness points of departure are shown in Figure 5A. For PFOA, we chose the frequentist Hill v1.1 model because it has the lowest AIC and the highest *p*-value (though still <0.1). All the models were deemed questionable. For the Hill model, constant variance test failed (test two *p*-value < 0.05) NS (0 dose): N is described in Figure 1. PFOA: four biological experiments. N = 8 for each dose except 100 uM where N = 6 for day 3 confluence fold change in Panel 6A, all of Panel 6B and Panel 6C. (a) are the NOAEL, (b) are the LOAEL, and (c) are significantly different from the 0 dose but not the LOAEL. Fig.6A: (b) have *p* < 0.0001 for day 1 and *p* < 0.001 for days 2 and 3; (c) have *p* value ranging from <0.005 to <0.0001. Fig.6B: (b) have *p* < 0.05, (c) have *p* ranging from <0.05 to <0.0001. Panel 6C: BMD = 483.8nM, BMDL = 278.3 nM.

PFOA induces dose-dependent suppression of lagging indicator confluence and leading indicator S-G2-M-phase cell cycle progression measured by fed/unfed peak fold change in green fluorescence. Analyses of the stimulation indices from the line graphs, now graphed as histograms, also show that PFOA suppresses accumulated growth as a lagging indicator measured by confluence (Figure 6A ) and suppresses entry into the S-G2-M phase after medium change as a leading indicator ( Figure 6B) predicted during ESC culture with LIF. In Figure 6A, Tfinal confluence means decrease with dose, but a second measure, the direness index, measures the first hour of departure of significantly decreased confluence compared with NS, 0 mM PFOA dose. In general, the green fold change decreases as the dose of PFOA increases. The day 1 peak, occurring after the T24 feeding, shows a clearer trend and is less noisy than the day 2 peak which occurs after the T48 feeding. In Figure 6C, a benchmark analysis of the point of departure was used to assess the dose of significant decrease compared with 0 mM sorbitol and normal stemness and such as Tfinal in Figure 6A, is accompanied by the direness significant point of departure for diminished confluence.

**FIGURE 7:**
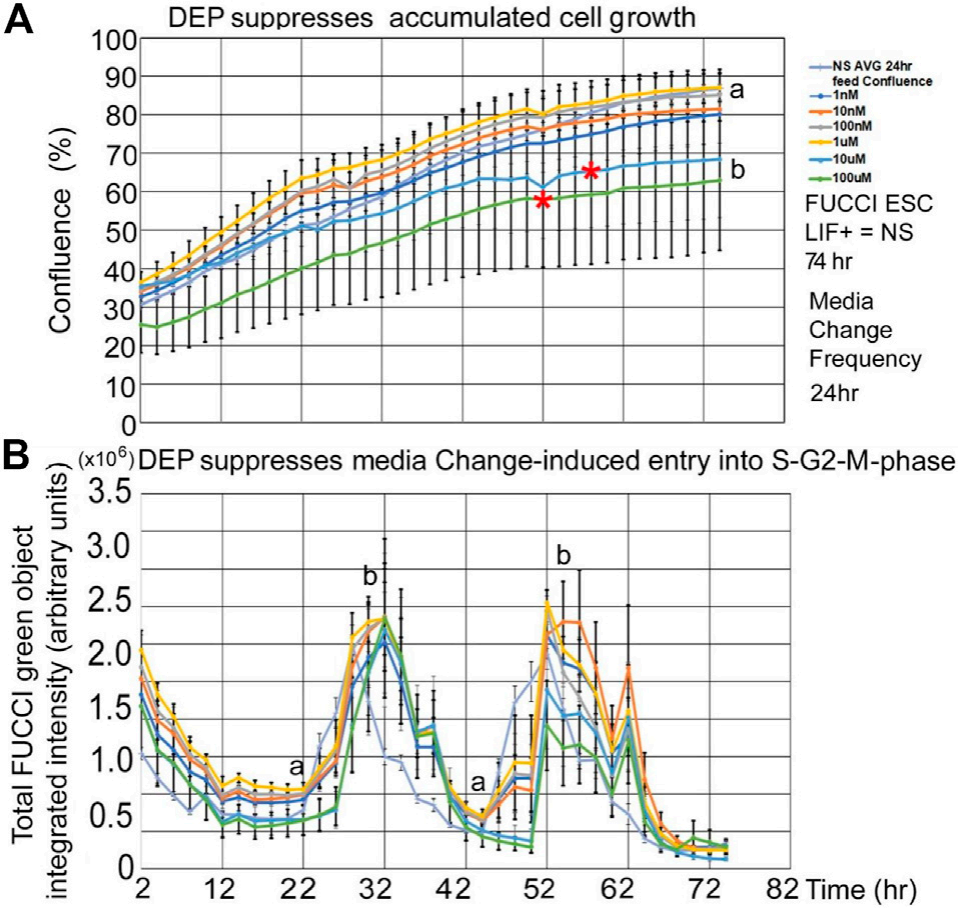
DEP suppresses accumulated growth as a lagging indicator measured by confluence and suppresses entry into the S-G2-M phase after medium change as a leading indicator predicted during ESC culture with LIF. FUCCI ESCs are cultured and assay as shown in Figure 1, but the indicated doses of DEP were added to LIF + media. **(A)** Confluence was measured in time-lapse every two hours in the live imager as described previously but here doses of 0 DEP and 1–100nM and 1–100uM DEP were used in LIF + culture. Red asterisks show when confluence starts to significantly differ from NS. For higher doses, the divergence points are realized earlier (T58for 10 uM and T52 for 100 uM). The rest of the doses are not significantly different than NS at T74. **(B)** Simultaneously, confluence green fluorescence was measured in time-lapse every two h in the live imager. Green intensity increases (cells enter the S-G2-M phase) after cells are fed at approximately T24 and T48. Increasing dose of DEP suppresses the number of cells in the S-G2-M phase, suppressing peaks. Offsets in the timing of the zero dose and the rest of the curves are due to feeding differences between experiments. NS (0 dose): N described in Figure 1. DEP: three biological experiments, N = 6. (a) is statistically different from (b).

**FIGURE 8:**
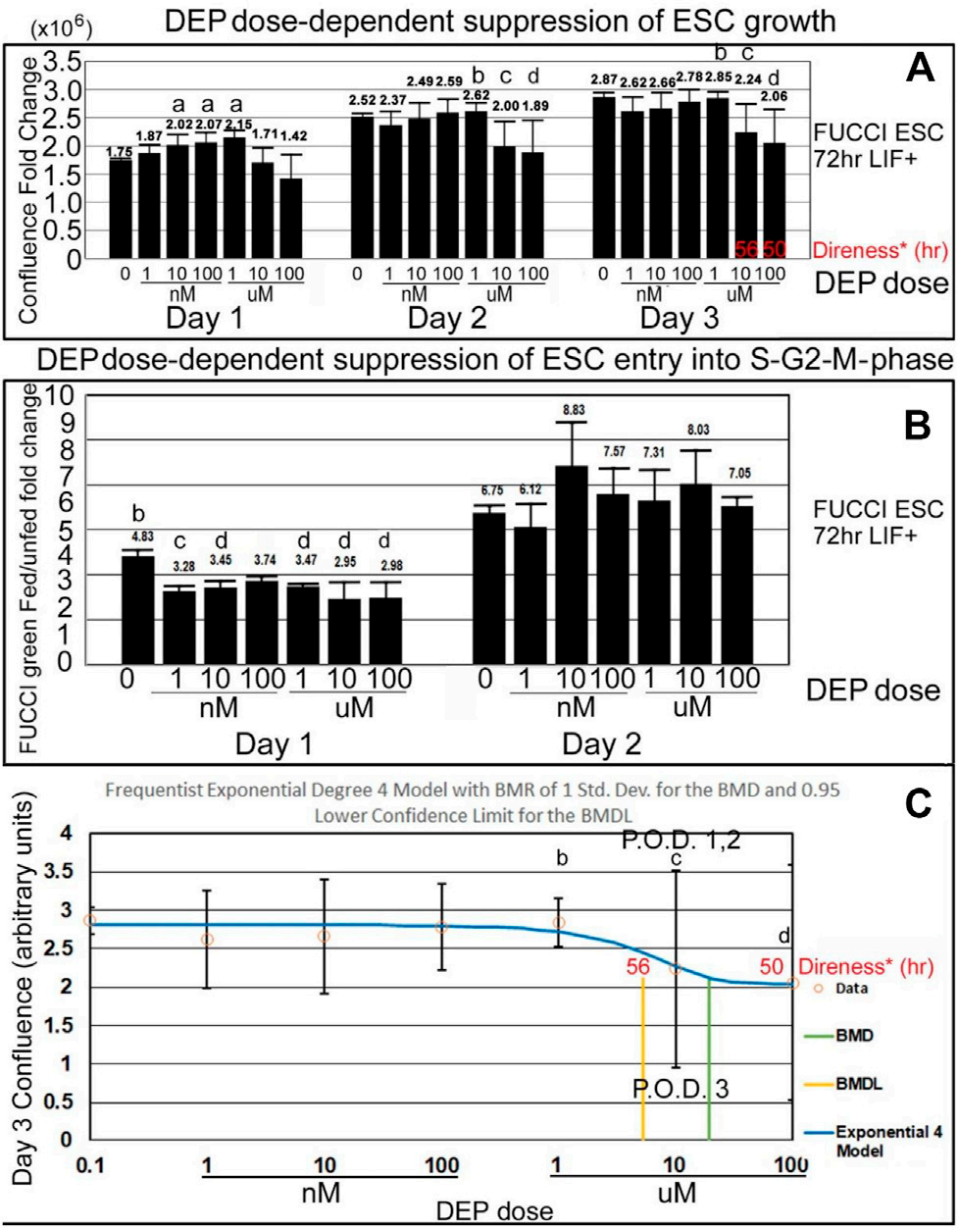
DEP suppresses accumulated growth as a lagging indicator measured by confluence and suppresses entry into the S-G2-M phase after medium change as a leading indicator predicted during ESC culture with LIF. **(A)** FUCCI ESCs are cultured and assayed as shown in Figure 4 , and data shown in Figures 7A,B are further analyzed for dose-dependent suppression of confluence and entry into the green S-G2-M-phase of the cell cycle. Confluence FC from timepoints on days 1, 2, and 3 were compared to time zero (T26/T0, T52/T0, and T74/T0), for DEP doses shown. As data of the confluences of the wells before the first feeding of the toxicant are not known, T0 used was the first time-lapse recorded (T2) for NS. **(B)** The amount of green intensity at the peak after feeding and the nadir before feeding are rationated. In general, the green fold change decreases as the dose of DEP increases. **(C)** Day 3 confluence FC from **A** was analyzed, using the Benchmark dose as point of departure. For DEP, we chose the frequentist Exponential degree 4 v1.1 model because it has the lowest AIC and the highest *p*-value (though still <0.1). All the models were deemed questionable. For the Exponential 4 model, the constant variance test failed (test two *p*-value < 0.05). Also, BMD/BMDL ratio >3 and modeled control response std. dev. >|1.5| actual response std. dev. NS (0 dose): N is described in Figure 1. DEP: N is described in Figure 7 . NOAELs are shown with (b) and LOAELs are shown with (c). Growth during the first day is significantly faster than NS for some intermediate doses (a) (*p* < 0.05). Day 1 peak, occurring after the T24 feeding, shows an early reduction in green FC at 1 nM (*p* < 0.01). This LOAEL is not replicated in FC for day 2 peak. (d) are doses that are significantly different from NS but not the LOAEL (A: *p* < 0.05, B: *p* ranges from <0.05 to <0.01) C: BMD = 19.2 uM, BMDL = 5.29 uM.

DEP suppression of confluence increases and fed/unfed peak green entry into the fluorescent green S-G2-M phase is lower in magnitude than PFOA but higher in sensitivity. DEP suppresses accumulated growth as a lagging indicator measured by confluence and suppresses entry into the S-G2-M phase after medium change as a leading indicator predicted during ESC culture with LIF (Figures 7A,B). DEP causes dose-dependent decrease in confluence and suppression of day 1 fed/unfed fold change entry into S-G2-M-phase cell cycle progression. Similar to PFOA, DEP causes dose-dependent decrease in confluence, but these are trends and are significant at higher doses (Figure 8A). However, the dose-dependent suppression of fed/unfed peak green entry into the S-G2-M phase was extremely sensitive on day 1 with a LOAEL at 1 nM (Figure 8B).

**FIGURE 9:**
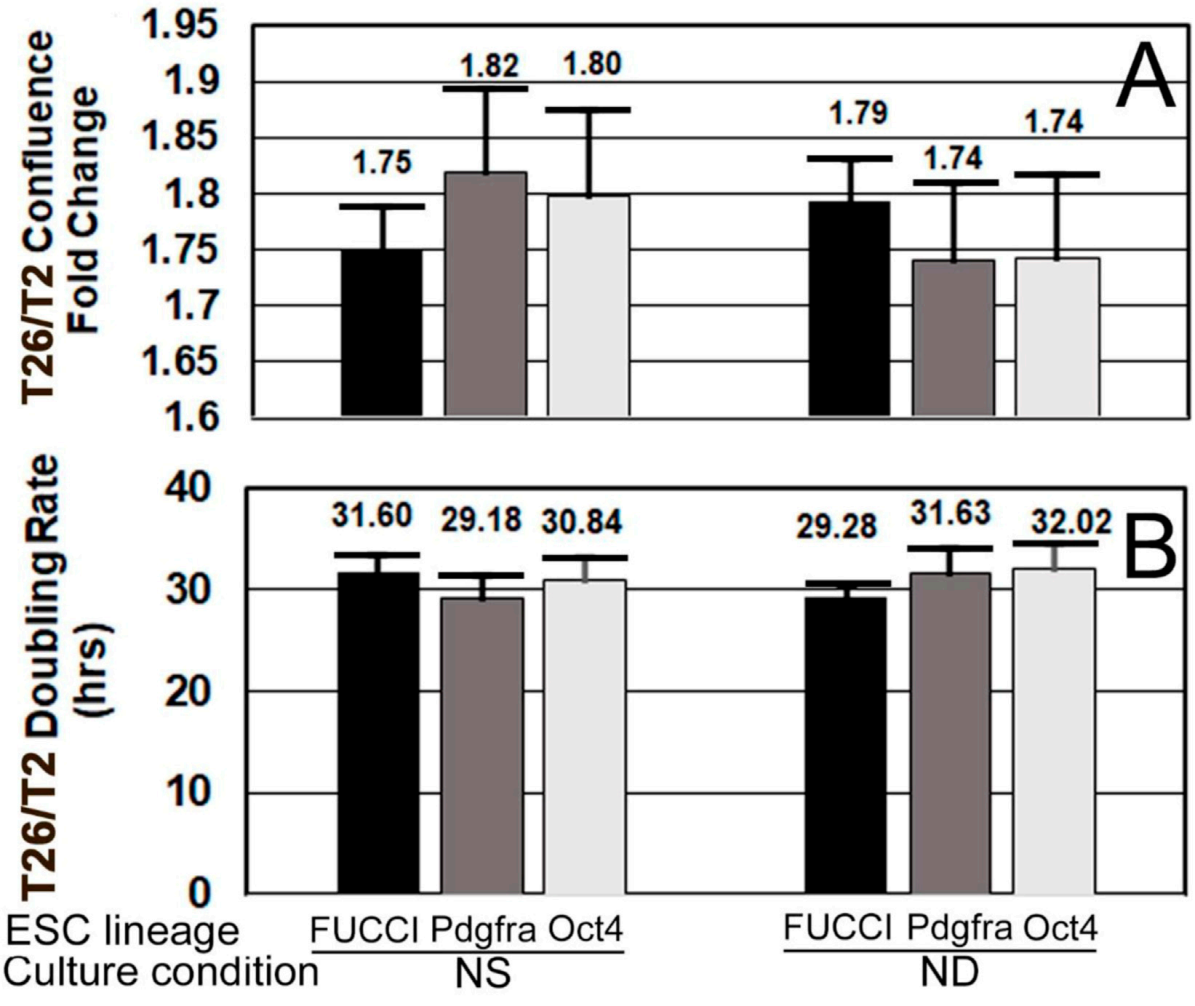
FUCCI and two other ESC lines have similar growth rates and doubling times when cultured in normal stemness (NS, LIF+) or normal differentiation (ND, LIF-) for one day. **(A)** FUCCI, Pdgfra, and Oct4 ESC were cultured and assayed as shown in Figure 4, except confluence increase and doubling rate were both measured in the first 24 h when rate of increase was highest and not as affected by contact inhibition. Confluence fold change from timepoints T2 to end of day 1 is shown. **(B)** During the culture of the same three ESC lines from T2–T26, change in confluence was used to compute the doubling rate. Six independent biological experiments for FUCCI NS (N = 26) and ND (N = 17). Four independent experiments for Pdgfra NS and ND (N = 8 for both).Five independent experiments for Oct4 ESC for NS and ND (N = 10 for both). There were no significant differences (*p* > 0.05) between cell types for NS or ND.

To confirm that FUCCI cells were performing normally, their confluence fold changes and doubling rates were compared with those of Pdgfra-GFP and Oct4-GFP cells. All three had statistically similar doubling rates between NS and ND culture after one day (Figures 9A,B).

## Discussion

Here, we report that FUCCI ESCs report stress-diminished growth in three ways, two of which are novel. First, the suppression of accumulated cells at Tfinal looks backward at stress effects through culture, and both PFOA and DEP suppress ESC accumulation albeit at different doses and levels of suppression. Second, the prediction of future cell growth can be done early in culture before contact inhibition obscures the growth of fastest growing stress-free controls. This predictive assay is based on the suppression of the progression into the green S-G2-M phase of the cell cycle after medium change relieves G1 delay. Third, live imaging provides a time lapse report of when the first significant adverse suppression of ESC accumulation occurs compared to lesser stress doses. The first two methods are based on the comparisons of stimulation indices, but the third method is based on “direness” indices which we have previously established that it can be a more accurate and less artifact-prone method to detect and quantify stressful hypoxic exposures in stem cells (Zhou et al., 2011; Bolnick et al., 2017; Yang et al., 2017b). Taken together, PFOA produces adverse effects (LOAEL and BMDL) faster and at lower doses than DEP for cumulative growth effects–like confluence (Table 1).

**TABLE 1:** Summary of dose- and time-dependent effects of toxicant stress on FUCCI-ESCs.

Sensitivity; accumulation						
	NOAEL (nM)	LOAEL (nM)	BMDL (nM)	IC50 (nM)		
T74** **h/T2 FC confluence (day 3)	—	—	—	—	—	—
** PFOA**	1	**10**	278	1747	—	—
** DEP**	1,000	**10,000**	5,294	5,022	—	—
T52h/T2 FC confluence (day 2)	—	—	—	—	—	—
** PFOA**	1	**10**	350	2,382	—	—
** DEP**	1,000	**10,000**	5,684	5,298	—	—
T26h/T2 FC confluence (day 1)	—	—	—	—	—	—
** PFOA**	1,000	10,000	1,464	8,176	—	—
** DEP**	none	none	51,006	58,632	—	—
**CONCLUSION: ESCs are 1000-fold more sensitive to PFOA > DEP**						
**MAGNITUDE; accumulation**						
	**Maximum confluence: 0M (72 h)**	**Minimum confluence: 100 nM (72 h)**	**Max/Min** **FC**	—	—	—
** PFOA**	2.87	**0.16**	**18FC**	—	—	—
** DEP**	2.87	**2.06**	**1.4FC**	—	—	—
**CONCLUSION: ESCs undergo a 13-fold greater confluence loss to PFOA > DEP**						
** SPEED; accumulation**						
Direness index: First significant suppression of confluence	**Earliest adverse effect level time**	—	—	—	—	—
** PFOA**	**2 h, 100 uM**	12** **h, 10** **uM	36** **h, 1** **uM	54h, 100 nM	42** **h, 10 nM	58** **h 1 nM
** DEP**	**52 h; 100 uM**	58** **h, 10** **uM	—	—	—	—
**CONCLUSION: ESCs respond 26 times faster to PFOA > DEP**						
**Sensitivity; re-entry into the S-G2-M-phase**						
	**NOAEL (nM)**	**LOAEL (nM)**	**BMDL (nM)**	**IC50 (nM)**		
Fed/unfed green suppression day 1						
PFOA	100	1,000	240	972		
DEP	0	1 nM				
Fed/unfed green suppression day 2						
PFOA	10,000	100,000	25,967	41,294		
DEP	none	none	104,636	113,692		
**CONCLUSION: ESCs decrease cell cycle entry 1000-fold more on day 1 DEP > PFOA**						
But this reverses on day 2						
**MAGNITUDE; re-entry into the S-G2-M-phase**						
	Day 1 fed/unfed green FC NS	Day 1 fed/unfed green FC 100uM	100uM/NS FC			
** PFOA**	4.83	**1.25**	**3.9FC**	**(∼4FC)**		
** DEP**	4.83	**2.98**	**1.6FC**			
**CONCLUSION: ESCs undergo a 2.5-fold greater confluence loss to PFOA > DEP**						

1 SUMMARY PFOA works faster and at lower doses for adverse effects than DEP for cumulative growth effects like confluence. (in DISCUSSION, summary of paragraph 1)

2 FIRST WAY. By dosage, PFOA has a 1000-fold more sensitive LOAEL doses than DEP for suppressing ESC accumulation (confluence) at day 3 and day 2 (that is., 10 nM for PFOA and 10,000 nM for DEP, respectively) and an infinitely more sensitive LOAEL at day 1 than DEP.

3 SECOND WAY. In terms of speed, PFOA is 26 times faster than DEP for producing a time-dependent LOAEL dose at 100 uM, six times faster at 10 uM, and infinitely faster at 10 nM–1uM than DEP for cumulative adverse effects on confluence. INTEGRATED

4 THIRD WAY. For instantaneous predictive growth effects, for FUCCI ESCs that are green and will divide in the future), DEP has effects at low doses on day 1, but the cells recover, and DEP has no significant suppressive effect on day 2. Thus, DEP suppression of future growth on day 1 is reversible by day 2.

5 INTERESTINGLY**,** the instantaneous fed/unfed state analysis picks up something that the cumulative growth stimulation index and direness index do not pick up, an apparent extremely sensitive (LOAEL at lowest dose: but reversible effect by DEP on day 1 that is not seen with PFOA which does not reverse.

First, analysis of the differences in relative ESC sensitivity to PFOA or DEP suppression of accumulation: PFOA has 1000-fold more sensitive LOAEL doses than DEP for suppressing ESC accumulation (confluence) at day 3 and day 2 (that is, 10 nM for PFOA and 10,000 nM for DEP, respectively) and an infinitely more sensitive LOAEL at day 1 than DEP (that is, 10,000 nM for PFOA and no BMDL dose for DEP, respectively) (Table 1 ). In the case of PFOA, the LOAEL dose for cell accumulation decreases from 10uM to 10 nM with time progression from one to three days. As more cells accumulate in the NS/zero dose exposure, the accumulative stress exposure is detected at lower doses. The stimulation index of FC suppression of accumulation comparing 100uM PFOA and 0uM/NS (that is, the comparison with the maximal FC) is about 18 FC on day 1, 30 FC on day 2, and 18 FC on day 3. For DEP, it is much smaller, about ∼1.2 FC, ∼1.3 FC, and ∼1.4 FC for days 1, 2, and 3, respectively. Toxicity can be thought of as a product of the lowest dose that a LOAEL is detected at and the magnitude of the toxic effects. Here, the magnitude is high for the suppression of cell accumulation by PFOA and low for DEP.

Second, analysis of the differences in relative ESC sensitivity to PFOA or DEP by the speed of growth suppression: Use of the live imager produces time-lapse line graphs for each dose of PFOA and DEP, and one can test for which time point a dose becomes significantly less in cell accumulation than NS at that time point. In terms of speed, PFOA is 26 times faster than DEP for producing a time-dependent LOAEL dose at 100 uM (that is, two h for PFOA and 52 h for DEP; Table 1 at the bottom), six times faster at 10 uM, and infinitely faster at 10 nM–1uM than DEP for cumulative adverse effects on confluence. Thus, for PFOA the time-lapse point of departure is much earlier. The highest dose at 100 uM PFOA has significantly lowered cell accumulation at two h after Tzero, 10 uM at 12 h, 1 uM at 36 h, and so on. Is it reasonable to see early time point effects? Diminished cell growth is not the cause of lower accumulation at the highest dose—100 uM PFOA; since fewer cells are present than at Tzero, death must account for this first point of departure. But for higher doses, that is, <100 uM, there are positive increases in accumulation and some effects as early as 12 h. This is consistent with the effects of stress enzymes that diminish Oct4, Sox2, Nanog, and Rex1 protein levels by 4 h and are activated highly by 1 h of hyperosmotic stress which slows growth rapidly (Slater et al., 2014). Although Oct4, Sox2, and Nanog protein levels return to normal by 24 h of hyperosmotic stress, Rex1 is maintained at lower levels by Mek1 at 1–4 h and SAPK at 24 h, respectively (Slater et al., 2014). Yet, the effects of Rex1 suggest that some stemness features have decreased within 2–4 h, and these decreases persist. Rex1 and Oct4 control the metabolic programs of stemness that control growth rates (Frum et al., 2013; Son et al., 2013) and hyperosmotic stress-forced differentiation to the extra-embryonic endoderm (also known as XEN) (Frum et al., 2013; Slater et al., 2014; Li et al., 2019). Time-lapse direness assayed here is also likely to be based on the large programmatic effects of these rate-limiting stemness factors. So, it can be concluded that hyperosmotic stress at 300 mM sorbitol or similar effects of 10–100 uM PFOA have early higher effects both on growth and stemness at higher doses and lesser effects at longer durations, as indicated by data here and in previous reports where the metabolism and stemness change over time. It will be important to determine using transcriptomic analysis if PFOA also induces changes in metabolism and stemness that are induced by hyperosmotic stress (Abdulhasan et al., 2021a; Abdulhasan et al., 2021b; Abdulhasan et al., 2021c).

The third analysis is the magnitude of suppression by PFOA or DEP on accumulative growth by confluence or suppressed entry into the S-G2-M-phase on day 1–2 feeding. By the suppression of stress of re-entry into the green S-G2-M-phase after medium feeding, PFOA has 10-fold greater effects. And by the analysis of accumulation, PFOA causes 13 greater effects than DEP. This delay in G1 by 24-h feeding was not anticipated, and the analysis of re-entry and its suppression by stress produced interesting outcomes. Unlike the assay of accumulation, re-entry after feeding into the S-G2-M-phase predicts future growth and its suppression by environmental toxicants. DEP has small but significant suppressive effects at low doses on day 1, but the cells recover, and DEP has no significant suppressive effect on day 2 on fed/unfed FC increase in green cells. Thus, DEP suppression of future growth on day 1 is reversible by day 2. Interestingly, the instantaneous fed/unfed state analysis picks up something that the cumulative growth stimulation index and direness index do not pick up, an apparent extremely sensitive (that is, the BMDL at lowest dose of 1 nM) but reversible effect by DEP on day 1. This reversibility is unique to DEP and was not seen with PFOA which does not completely reverse at exposures days 2 and 3. In contrast, the NS to 100 uM FC on day 1 for PFOA suppression of S-G2-M phase re-entry is ∼4FC and for DEP only ∼1.6 FC, respectively. For the suppression of entry into the cell cycle after the G1-phase by stress on day 1 and 2, there is 10-fold more suppression by PFOA than DEP (Summary Table 1; ∼4/∼1.64 = 2.5). Thus, early suppression of re-entry by DEP is exceedingly small at the highest dose and is very sensitive, occurring at the lowest dose, but is reversible. It will be interesting to test for long-term changes in anabolic and epigenetic transcriptomes in reversible DEP vs irreversible PFOA exposures to find whether brief reversible stress episodes have long-term effects.

For DEP, a different story emerges compared with PFOA for cell accumulation as would be predicted by the lesser effects of DEP on the suppression of future growth discussed above. There is a much shallower suppression of the magnitude and direness of growth accumulation by DEP compared to PFOA. For DEP, the suppression is lower in magnitude than PFOA. And points of departure for decreased accumulation for DEP are 58h at 10 uM and 52 h at 100 uM compared with 12 and two h for PFOA, respectively. Another article says that for some cases, the “direness” index, reported from the speed of the stress response to toxicants, is more accurate and artifact-free than the standard stimulation index (Yang et al., 2017b). For DEP, the NS to 100 uM FC for accumulation is only 1.23 FC, 1.33 FC, and 1.39 FC on day 1, 2, and 3, respectively, and FC suppression of entry into the S-G2-M-phase for the highest dose on day 1 is only 1.62 FC. The growth rate for DEP is less suppressed than for similar doses of PFOA despite DEP having a lower day 1 LOAEL for the suppression of entry into the S-G2-M phase. This may be because the green suppression is only on day 1 and it reverses so that overall, the cell growth is not suppressed by a large amount.

It is uncertain whether there is a concern that PFOA may be toxic to the ESC lineage in the early first trimester in humans. There are several reports that PFOA has no effects on pregnancy outcomes (Stein et al., 2009; Savitz et al., 2012), preterm birth (Gardener et al., 2021), and gestational weight gain (Marks et al., 2019). But, our FUCCI ESC assay models early first trimester events, when others reported exposures in the first trimester (Wikström et al., 2021). Increasing PFOA exposure from the 25th to 75th percentile increased miscarriage by 50%. In mice, PFOA led to significantly increased early pregnancy loss (Lau et al., 2006). In one report, PFOA exposure alone in the first trimester did not affect later pregnancy, but PFOA and cigarette smoke co-exposure negatively affected fetal growth at a later stage (Mamsen et al., 2019). A study of human embryonic and fetal tissues obtained in Denmark from 2014–15 found that by the end of the first trimester, PFOA was the second highest PFAS family member in maternal blood, placenta, and many fetal organs with PFOS being the highest (Mamsen et al., 2019). Thus, PFOA crosses the placenta early and accumulates in the fetal organs. First trimester exposures to PFOA and PFOS are associated with an approximate 10% decrease in fecundity (Vélez et al., 2015). Taken together, the data suggest that first trimester PFOA exposures cross the placenta and enter fetal organs and are associated with increased pregnancy loss or decreased fetal growth.

Several PFOA exposures are in the range of the BMDL effects that are reported here. The highest workplace exposure was at the Cottage Grove MN 3M PFOA synthesis plant at about 210 uM (Kudo and Kawashima, 2003) whereas the highest of the three levels of blood PFOA stratified in workers there was >28 uM. In the environment, point exposures such as ground water in Little Hocking OH were 1 uM (Vestergren and Cousins, 2009), and worldwide average blood exposures were 5–20 nM (Vestergren and Cousins, 2009). The highest level was found in cord blood, and thus the fetus or embryo was also 20 nM (Negri et al., 2017), but there are only a few reported cord blood levels. Thus, the BMDL doses reported to affect ESC accumulation of cell cycle progression are within the limit of maternal blood and cord blood exposures.

DEP may be toxic to the ESC lineage in the early first trimester in humans. DEP harms animal and human reproduction (Radke et al., 2019; Dutta et al., 2020; Weaver et al., 2020). DEP increases the body weight of F1 after *in utero* exposure in rodents, decreased the growth rate of cord blood–derived hematopoietic stem cells, and decreased BMI in humans. DEP and DEHP, another phthalate, have been associated with increased pregnancy loss and recurrent pregnancy loss (Messerlian et al., 2016; Liao et al., 2018). Thus, like PFOA, DEP may affect early first trimester events. Evidence suggests that DEP cross the placenta and affect the embryo and its ESC lineage. Phthalates affect the first trimester placenta by changing its transcriptome and methylome (Grindler et al., 2018). Although DEP was not studied, many phthalates elevated in first trimester maternal blood are also associated with DNA methylation changes in the offspring (LaRocca et al., 2014) and pregnancy loss (Mu et al., 2015), suggesting *trans*-placental phthalate effects. DEHP is found in cord blood and is associated with lower birth weight (LaRocca et al., 2014). Thus, phthalates, in general, and DEP, specifically, cross the placenta, are detected in cord blood, and have first trimester effects that should be predicted by screening cultured ESCs that emulate first trimester embryogenesis.

FUCCI ESCs in the studies here are consistent with yield outcomes that are then consistent with previous reported outcomes. We report the slowing of the cell cycle here with environmental toxic stressors, consistent with the slowing of the cell cycle using positive control hyperosmotic stress reported previously (Slater et al., 2014; Li et al., 2016a; Li et al., 2016b; Li et al., 2019). Interestingly, as the cell cycle is slowed down experimentally with hyperosmotic stress, it has been reported that G1 delay is associated with increased endoderm differentiation and less neuronal differentiation (Coronado et al., 2013; Pauklin and Vallier, 2013), and when we use stress to slow the cell cycle, with suggested G1 delay here, we have previously shown that the extra-embryonic endoderm increases (Slater et al., 2014; Li et al., 2019; Abdulhasan et al., 2021a; Abdulhasan et al., 2021b) and GO groups for neuroectoderm decrease (Abdulhasan et al., 2021b). Thus, it will be important to test whether experimental stressors also override stemness and proliferation signals from LIF and imbalance forced differentiation similarly to hyperosmotic stress.

Unlike previous growth measurements of stressed ESCs, time-lapse measurements given here were done by the analysis of confluence. But, the IncuCyte Zoom live imager reports confluence-based doubling times of 29–32 h for three transgenic muESCs (Oct4-GFP, Pdgfra-GFP, and FUCCI) whereas previous reports used synchronized serum-starved and refed muESCs at 11 h (Stead et al., 2002), 10–14 h (Pauklin and Vallier, 2013), 10–30 h (Nakai-Futatsugi and Niwa, 2016), and 10 h (Sakaue-Sawano et al., 2017). The discrepancy is likely due to the live imager use of confluence of an ESC epithelium that is squamous near Tzero and columnar near Tfinal and that the higher density of the columnar is missed by the confluence-based cell number and doubling rate estimates. These discrepancies can be remedied by use of a technique that counts the number of cells instead of measuring confluence. For this initial report, the conclusions reached should be valid but would be more accurate with object-based, rather than confluence-based, estimates of growth rates.

Interestingly, the use of a live imager to monitor cell cycle progression of FUCCI ESCs has not been reported, and cell cycle timing is usually evaluated by synchronizing the cells by serum starvation, (Zielke and Edgar, 2015) and not partially synchronized by medium change as performed here. Exit from the green S-G2-M phase after standard daily feeding protocols for ESCs has not been reported. This exit or abnormal delay in non-green G1 does occur with serum starvation prior to cell cycle synchronization, which occurs when G1 phases as short as one h in FUCCI-ESCs have been reported, but serum starvation is delayed for 24 h (Sakaue-Sawano et al., 2017). The delay in the non-green G1 phase could be due to increased lactate acidosis, due to aerobic glycolytic anabolic metabolism enabling rapid ESC cycles, or to depleted nutrition. Lactate acidosis is a problem as a report suggests that pluripotent stem cells which grow at high densities are hindered by increasing acidosis, and growth is enabled by buffering acid with sodium bicarbonate (Liu et al., 2018). We did not use bicarbonate but found that more frequent medium change at 12 h suppressed the fed/unfed green peak of the S-G2-M phase progression and smoothed the growth curve compared to 24-h feeding frequency. That medium change did not enable increased growth may be due to confounding variables such as the use of a confluence-based growth assay or feeding with media that were not pre-equilibrated and pre-saturated with 5% CO_2_, the standard culture level of CO_2_.

Current regulatory guidelines from the Organization for Economic Co-operation and Development (OECD) are OECD 443 “Extended One-Generation Reproductive Toxicity Study and OECD 414 “Prenatal Developmental Toxicity Study” which are performed in gestational rodents and lagomorphs. However, OECD also hosts an online adverse outcomes pathway analysis forum established by an article on the AOP https://www.oecd.org/chemicalsafety/testing/adverse-outcome-pathways-molecular-screening-and-toxicogenomics.htm (Villeneuve, Crump et al., 2014). The current and relatively new approach to regulatory approval is to contribute an AOP at the OECD webpage for the AOP knowledge base (AOP-KB) https://aopkb.oecd.org/background.html to provide international harmonization of development in collaboration with the applicant and with a national committee for “validation of alternate methods” (VAM) to *in vivo* testing. Many countries have (VAM) committees. In the US, the VAM is Interagency Coordinating Committee (ICC) https://ntp.niehs.nih.gov/iccvamreport/2015/about/iccvam/index.html which collaborates with the OECD to integrate national and international development efforts with the inventor and developer of the alternate methods.

Taken together, the data here suggest that several measurements of normal and toxicant-modulated growth of ESCs are enabled by live imager time-lapse data for 1) confluence-based accumulated growth assay, 2) predictive cell cycle progression assay of the suppression of cell cycle progression which diminishes future accumulation, and 3) direness index (Yang et al., 2017b)–based analysis of earliest detection of suppressed accumulated growth compared with lower doses. The use of these assays should be applied to more toxicants to inform which toxicants endanger ESCs at lowest levels and fastest endpoints. Additional endpoints of transcriptomic, epigenomic, and metabolomic nature should be used to complement growth analysis and inform teratogenic and transgenerational effects.

## Data Availability

The original contribution presented in the study is included in the article/Supplementary Material; further inquiries can be directed to the corresponding author.
